# Denis Mukwege: healing the survivors of sexual violence

**DOI:** 10.2471/BLT.24.030324

**Published:** 2024-03-01

## Abstract

Denis Mukwege talks to Gary Humphreys about treating the survivors of wartime sexual violence and driving global advocacy efforts to end to it.

*Q: How did you come to your focus on gynaecology and obstetrics?*

A: My initial vocation was to specialize in paediatrics, but I decided to focus on gynaecology and obstetrics when I saw how many women were dying giving birth in my country. The maternal mortality rate in the Democratic Republic of the Congo is one of the highest in the world. So, having graduated in medicine from the University of Burundi, I pursued my specialization at the University of Angers in France, then returned to my home province of South Kivu in the Democratic Republic of the Congo, to care for women facing severe gynaecological complications.

*Q: You were working at the Lemera Hospital in South Kivu when a massacre took place there in 1996. How did that experience shape your thinking and career?*

A: The massacre at Lemera hospital resulted in the death of at least 37 people, and as the medical director, I felt responsible for the safety of both patients and staff. So, it was, of course, a profoundly traumatic experience, but it only fuelled my commitment to advocate for peace and justice in the Democratic Republic of the Congo and around the world. I have come to see it as the beginning of an era of impunity for mass atrocities, and also as a symbol of the international community’s indifference in the face of such crimes. Everyone agrees that hospitals cannot be a target for violence, and that such grave breaches of international humanitarian law should be investigated and prosecuted, but in Lemera no one has ever been held accountable and the remains of the victims are still lying in mass graves.

*Q: Just three years after the Lemera massacre, you established Panzi Hospital in Bukavu, the capital of South Kivu. What were your aims in setting up that facility?*

A: Our primary aim was to fight maternal mortality by providing a safe place for women to give birth, but as it turned out the first patient to come in had been raped – raped and mutilated with extreme brutality. It was the first time we had witnessed such a barbaric act. We all thought we were dealing with an isolated incident caused by some deranged individual, but then many more cases came in, and we realized that the women and girls were survivors of the war that was still raging in the region. As a result of the patients we received, we were forced to move away from our original plan and specialize in reconstructive surgery of the female genitalia, as well as disabling gynaecological pathologies such as lower urogenital and digestive fistulas. Since 1999, we have helped to care for more than 80 000 survivors of sexual violence.

“We have helped […] more than 80 000 survivors.”

*Q: What drives conflict-related sexual violence?*

A: What drives people to use weapons? There is growing evidence that in most conflict situations, the perpetrators use rape to humiliate, dominate, spread fear, and displace targeted communities. These heinous acts are often committed in public, and not only affect the victim, but destroy the social fabric of communities and families through psychological trauma, shame and stigma.

*Q: What are the consequences of such violence and how they can they be addressed?*

A: The consequences for the survivor are multiple, and addressing them requires more than surgery. Survivors often have psychological trauma and may have difficulty sleeping, or wish to end their own life. In addition, many survivors are ostracized by their communities or even abandoned by their families. To address these different challenges, we have developed a holistic approach to treatment and rehabilitation, offering psychosocial support, and providing survivors with job skills training and educational opportunities to help them make a living and to support themselves independently. Lastly, because recognition that they are victims of a crime is very important to many survivors, we provide legal services, and make every effort to help them prosecute their perpetrators if they can be identified.

*Q: What is the typical pathway for a woman entering your system?*

A: The entry point is usually our medical and surgical services, but as soon as a survivor is admitted, she is automatically assigned a psychosocial assistant who will accompany her throughout her healing journey. The assistant sets out the different pillars of holistic care that we offer, and helps the survivor decide which, if any, she wants to choose. Survivors receive therapy both in the hospital and at our rehabilitation and training centre, Maison Dorcas, which is where survivors live after they are discharged but are still recovering, and while receiving job-skills training. They may also meet with their lawyers there, or at the legal clinic nearby.

Our support at Panzi continues with an accompaniment of our patients in their communities. Through mediation and networking, mobile teams from the Panzi Foundation, with the support of the local churches and nongovernmental organizations, also contribute to rebuilding family and community ties that have been shaken by the atrocities they have suffered. One of our core aims is to make the survivors agents of change in their communities, and to develop their leadership skills once they have found the right solutions to their physical and/or mental health challenges. They thus participate in the reconstruction of the social and economic fabric that underpins a community’s health. There are many examples of women who have managed to do this. For example, some have pursued medical studies, others have become human rights defenders or social workers, helping the most disadvantaged.

*Q: Does your approach risk ‘programmatizing’ sexual violence response, detracting from the provision of services across the broader health system?*

A: We do what we can to boost the national system. Panzi Foundation operates three One-stop centres – two in rural areas in Kivu province, and one in the capital city of Kinshasa. This model allows survivors to access the care they need all in one location, and avoids them having to repeat their story over and over. The Panzi Foundation also partners with existing health facilities to integrate holistic services into their medical care, combining medical treatment with psychosocial support, socio-economic reintegration opportunities, and legal services.

*Q: Have you sought to export your model. If so, to what effect?*

A: The Mukwege Foundation advocates worldwide for the provision of holistic care to be recognized as a human right for all victims, and to be integrated into national health-care systems around the globe. We are also developing South-South cooperation projects to duplicate this model of comprehensive care in other countries affected by conflict, notably in the Central African Republic, Guinea, Burkina Faso and Iraq. It’s also worth noting that SEMA (“speak out” in Swahili), the global network of victims and survivors to end wartime sexual violence, started with an international retreat of survivors organized by the Mukwege Foundation based in the Netherlands back in 2017. Today, SEMA gathers members from 26 countries, and enables survivors to take a leadership role, speaking and acting as experts at international advocacy and policy events. Bringing survivors from different generations, continents and cultures together to learn from each other is invaluable, allowing people to see their personal suffering in a wider context. Organized, global advocacy also provides opportunities to amplify the voices of survivor activists and to represent their perspectives at the international level.

“Impunity continues to prevail.”

*Q: What progress has been made in holding people accountable of sexual violence in war?*

A: Since the adoption of United Nations Security Council Resolutions 1325 and 1820, the link between sexual violence and international peace and security has been clearly established. The Rome statute, the basis for the International Criminal Court, codifies rape and other gender-based crimes as a war crime, a crime against humanity, or even a constitutive act of the crime of genocide. This is a major step forward, because in order to deal with a problem, it must first be recognized. Thanks to this evolution in the normative framework, no political or military leader can overlook the fact that it is contrary to international law to use rape and sexual violence as a weapon of war and terror. Nevertheless, impunity continues to prevail, including in my own country. We are therefore advocating for a national justice strategy to address this issue.

*Q: In September 2012, you gave a speech at the United Nations where you condemned the mass rape occurring in your country and criticized the Congolese government and other countries for not doing enough to stop it. One month after the speech, there was an attempt on your life and you went into exile for a year. How did you find the courage to return?*

A: As to my speech at the UN, I felt an obligation to testify in front of those who supposedly had the power to put an end to these mass atrocities. Shortly after, my daughters were held hostage, and unidentified armed men attempted to murder me and killed my guard. Despite calls from the Democratic Republic of the Congo authorities, the diplomatic community and the Office of the United Nations High Commissioner for Human Rights to promptly investigate, no genuine efforts were made and no one was ever held accountable. As to my decision to return to my country, I felt I had no choice.

*Q: You recently entered the political sphere as a presidential candidate in your national election. What is next for you after that experience?*

A: We will continue our mission to care for and rehabilitate the victims of war, as well as our advocacy efforts for peace, justice, and the eradication of the scourge of sexual violence committed as a weapon of war and terror, in the Democratic Republic of the Congo and throughout the world. The way to peace in the Democratic Republic of the Congo and the Great Lakes region passes through justice, and we are advocating for the adoption of a holistic national transitional justice strategy to prevent the recurrence of mass atrocities and promote the rule of law. We are therefore calling for the establishment of an International Criminal Tribunal for the Congo; a profound reform of the security sector; and the vetting of our institutions, truth-seeking mechanisms, and reparation programmes. I am also calling on the international community to pursue similar goals, mobilizing the resources that are needed to advance an accountability agenda. If these heinous crimes go unpunished, they will continue.

